# Finite element analysis of Kirschner wire fixation for lateral condyle fracture in children in the sagittal plane

**DOI:** 10.3389/fped.2023.1210493

**Published:** 2023-07-24

**Authors:** Huanan Bai, Qingda Lu, Xiaoju Liang, Xiaoming Wang, Yating Yang, Huan Wang, Jiaju Wang, Qiang Jie

**Affiliations:** ^1^ Pediatric Orthopaedic Hospital, Honghui Hospital, Xi’an Jiaotong University, Shaanxi, China; ^2^ School of Medicine, Medical College of Yan'an University, Yan'an, China

**Keywords:** lateral condyle fracture, finite element analysis, Kirschner wire, sagittal plane, children

## Abstract

**Objective:**

This study aims to find the optimal arrangement of the Kirschner wire (K-wire) in the sagittal plane for fixation of a pediatric lateral condylar humeral fracture (Milch type II) by using finite element analysis (FEA).

**Methods:**

A model of lateral condyle fracture in a 6-year-old boy was developed, and an *XYZ* coordinate system was established based on this model. The *YZ* plane was defined as the sagittal plane to investigate the impact of the angle formed by the first and second K-wires on stability. Two configurations were studied for each angle: parallel and divergent. Evaluation indicators included the maximum displacement of the fracture fragment and the maximum von Mises stress in the pins and bone.

**Results:**

The model with a −60° angle showed the best performance in both evaluation indicators. The parallel and divergent pin configurations had different performances in each group. The displacement results for negative angles were similar, and this result was better than those for positive angles.

**Conclusion:**

We successfully created a model of pediatric lateral condyle humerus fracture (Milch type II) and performed K-wire fixation with varying sagittal plane configurations, combined with FEA. Our findings demonstrate that the angle of −60° between the two pins in the sagittal plane provided the highest level of stability, with divergent configurations proving superior to parallel pinning at this angle.

## Background

Lateral condyle fractures are the second most common fracture in the pediatric elbow after supracondylar fractures, with a reported incidence between 10% and 15% in the literature. These fractures typically occur in children 4–10 years old with boys being more affected than girls (1, 2). The cause of the injury is related to one-sided arm support while the child is playing, and there are several types of fracture displacement such as Jakob, Milch, Finnbogason, Song, Weiss, Rutherford, Badelon, Lagrange, Hasler, Laer, Salter–Harris, and so on. Fractures with less displacement achieve good results with plaster or brace fixation, while fractures with greater displacement require operative treatment, including closed Kirschner wires (K-wires) and open reduction. The damage can result in fractures within the joint, and improper treatment can further impair both the appearance and function of the elbow joint in the long run. Increased risk of complications include elbow stiffness, cubitus varus, and cubitus valgus. Therefore, proper fixation is paramount to ensure optimal healing and preservation of joint function.

Although understanding of the fixation of lateral condyle fracture healing has recently increased, there are still complications such as nonunion or delayed union, growth disturbance, limited range of motion, residual deformity, and lateral condyle overgrowth that have been described (3). This is highly correlated with the use of fixed methods. K-wire fixation as a mainstream fixation, K-wires with tension band fixation, screw fixation (bioabsorbable or not), and screw combined with K-wire fixation have been successfully applied (4–8).

However, most of the studies limited the number of patients or solid models, and the finite element analysis (FEA) theory is less studied in this area (9). Current research predominantly investigates coronal plane wiring techniques, and the lack of sagittal plane wiring methods remain inadequately studied. Even though K-wire fixation has become mainstream in this field, both clinical and theoretical studies have been limited to coronal plane studies, and no sagittal plane study has been conducted. Based on the current theory and FEA, we have studied the angle of the K-wires on the sagittal plane to examine the stability of the K-wire fixation after the approval from the institution's ethical review committee.

## Materials and methods

### Computational modeling of the lateral condyle of the distal humerus

This biomechanical study was performed with the approval of our institution's ethical review committee (No. 202211002).

We obtained a normal computed tomography (CT) data from a 6-year-old boy who was evaluated for occult right elbow injury in our hospital. The Digital Imaging and Communications in Medicine (DICOM) data file was imported into Mimics 21.0 (Mimics 21.0, Belgium) software to manually segment the bone and ossification nucleus of lateral condyle of humerus and cartilage components based on the grayscale.

Given that the model built in Mimics 21.0 had a rough surface, we used Geomagic software (Geomagic 2013, USA) to denoise the model and convert it into a surface entity, and we obtained a cancellous bone by offsetting the cortical bone inward by 2 mm. We imported all the above structures into SolidWorks 2018 software (SolidWorks 2018, USA) for assembly and cut a fracture line passing through the middle of the distal humerus to simulate a lateral condyle fracture.

### Finite element model generation

We designed a fixed model for K-wire insertion in bone fractures using SolidWorks 2018, which includes two K-wires and the previously designed model. To adhere with the clinical standards, we purposely set the diameter of the K-wires to 1.6 mm.

**Figure 1:**
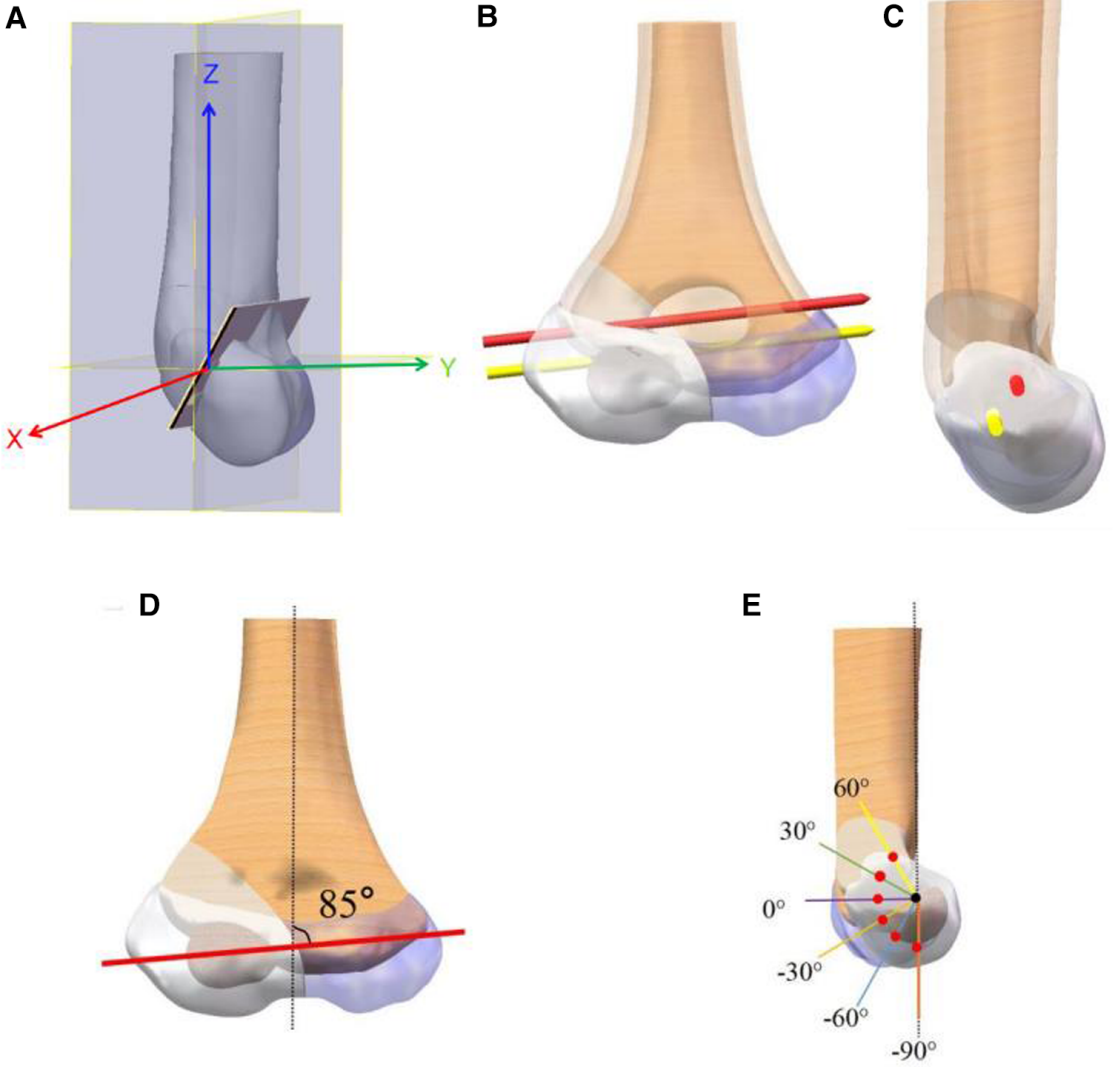
Schematic diagram of the coordinate system (**A**). (**B,C**) The coronal plane and sagittal plane of the −60° model, respectively. (**D,E**) The different model groups of coronal and sagittal positions.

We established a three-dimensional system of *XYZ* in SolidWorks, where the *Z*-axis is set to the longitudinal axis direction of the humerus from bottom to top. The corresponding *Y*-axis is defined from the back to the front direction of the fracture model, and the *X*-axis direction is from the inside to the outside (Figure 1). In order to better display in the sagittal plane, we defined the origin of the system to the center of the outer section of the first K-wire.

Through the above steps, we successfully designed a fixed model for K-wire insertion in bone fractures that strictly adheres to clinical standards and provided a coordinate system definition for studying the differences in K-wire placement angles.

On the coronal plane, the angle between the K-wire and the *X*-axis is set to 5° (9). On the sagittal plane, we divide the line connecting the two K-wires into six groups from 60° to −90°, each group including parallel and dispersed configurations. To ensure precise results, we used the 0° parallel configuration to fully open K-wires and apply this angle to the other groups. This ensured that the angle at which the K-wire is opened remains consistent across all scattered needle models (Figure 1).

### Finite element calculation and determination of stiffness

We imported all generated models into ANSYS Workbench 2019 (ANSYS 2019, USA) software for analysis. The material parameters of the model are shown in Table 1.

**Table 1:** The parameters of materials.

Part	Young's modulus (MPa)	Poisson ratio
Cortical bone	11,670	0.28
Cancellous bone	70	0.2
Ossific nucleus	70	0.2
Cartilage	15	0.45
K-wires	200,000	0.33

The material parameters of the K-wire were provided by a cooperative manufacturer, and other material parameters were obtained from existing literature (10, 11). If the maximum result was within the 95% range and there is no maximum stress point, we will continue with further research. The model was ultimately divided into tetrahedral-dominated elements, with consistent settings applied to all models to minimize discrepancies from the mesh division. For the −60° model, the elements count was 714,360 with 1,047,273 nodes.

**Figure 2:**
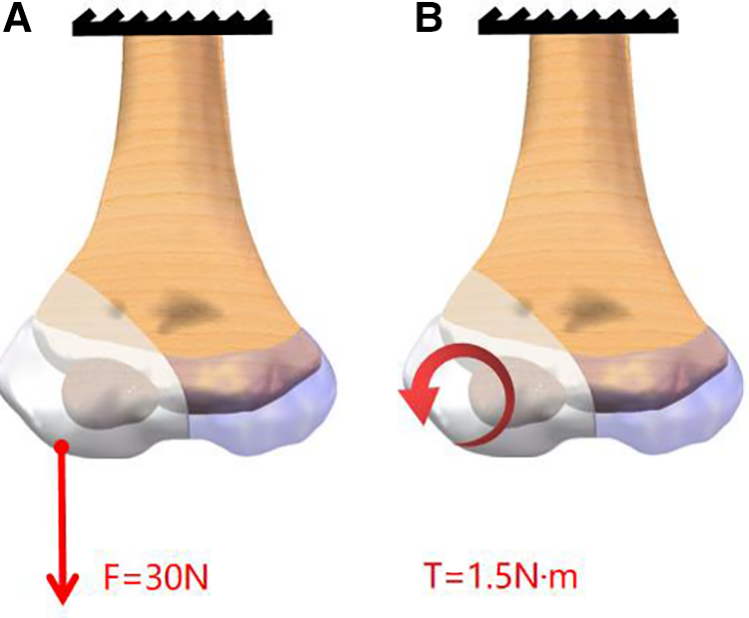
Schematic diagram of model loads. A force of −30 N parallel to the *Z*-axis was applied to the distal of the fracture block A torque of 1.5 N m with the *Y*-axis as the center to simulate the lateral collateral ligament on this position.

The proximal of all models was set to 0 degrees of freedom to restrict displacement. A force of −30 N parallel to the *Z*-axis was applied distally to the fracture block when simulating gravity. When simulating internal rotation, this area was set to a torque of 1.5 N m with the *Y*-axis as the center to simulate the lateral collateral ligament on this position (Figure 2).

We analyzed and recorded the maximum displacement of the humerus distal end, peak, and distribution of von Mises stress of the K-wire and bone for each model based on the results under different angles.

## Results

**Figure 3:**
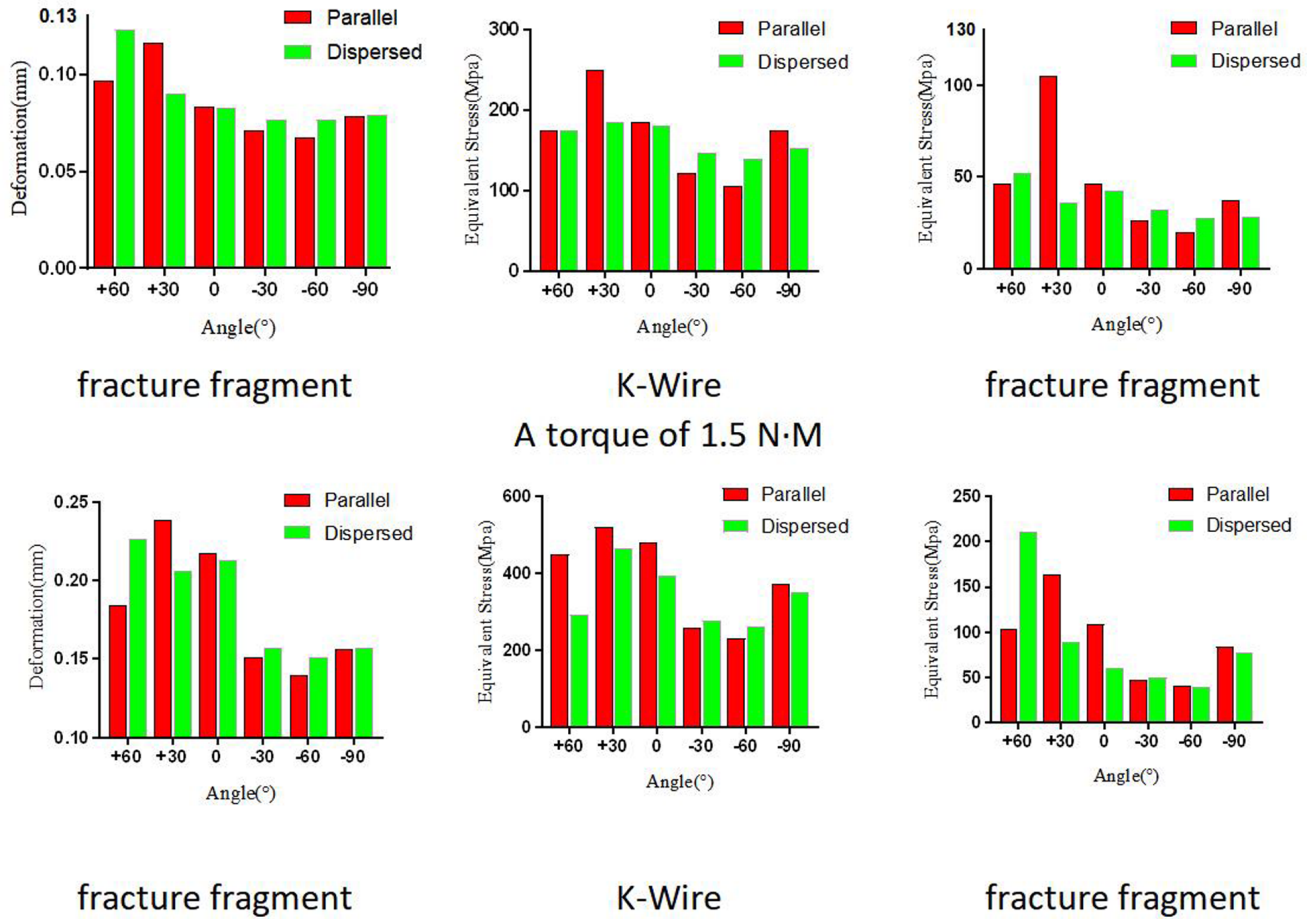
The displacement of fracture fragment, and the von Mises stress distribution of K-wires and bones in different angles and loads.

**Figure 4:**
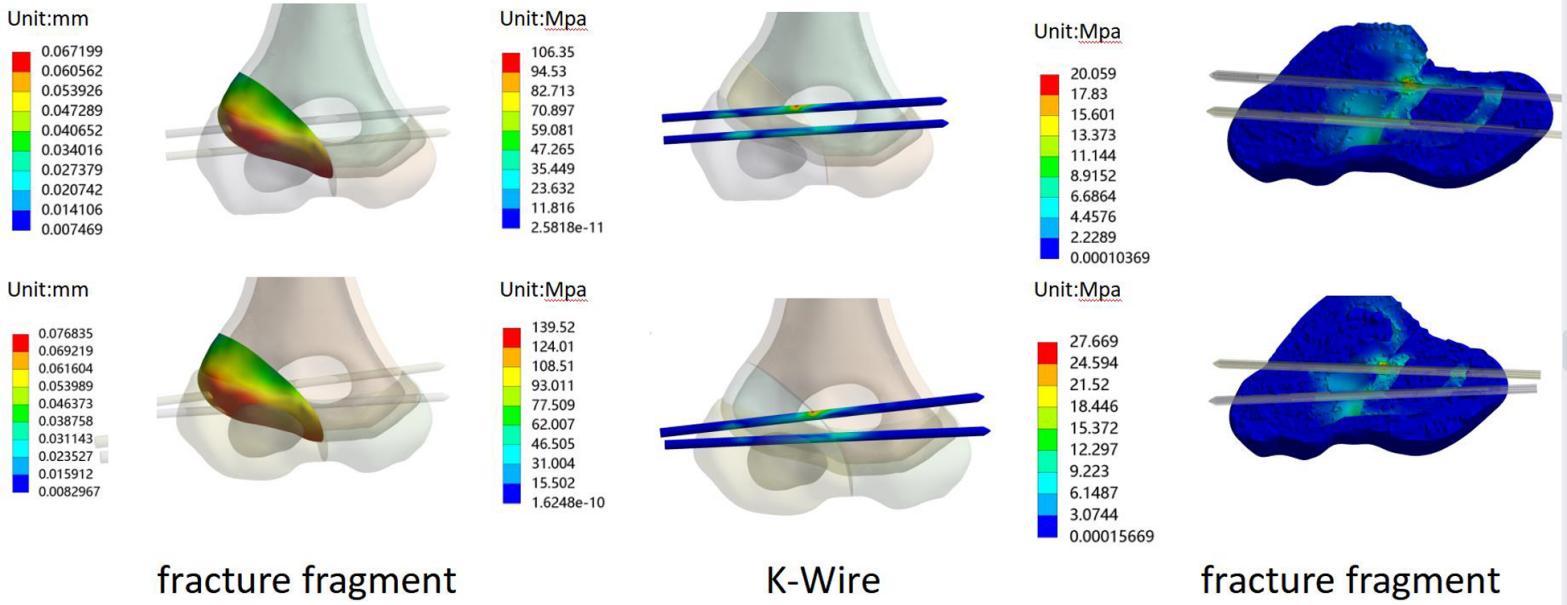
For the −60° model: the displacement of fracture fragment, and the von Mises stress distribution of K-wires and bones in force (up) and rotation (down).

Our results showed the displacement of the fracture fragment as well as the von Mises stress distribution of K-wires and bones. Figure 3 illustrates the relationship between the maximum values of each model under different loads. Figure 4 presents the displacement distribution of fracture fragments, and the von Mises stress distribution of K-wires and bones for the −60° model.

The peak displacement values for fracture fragments varied among the different fixed angles, indicating a difference in stability. At −60°, the peak displacement value was the smallest, with tensile forces and rotational conditions parallel to the K-wires being 0.067199 and 0.13972 mm, respectively, and for dispersed K-wires were 0.076835 and 0.15094 mm, respectively. The detailed distribution of displacement can be seen in Figure 3. The stress distribution of K-wires and bones was similar to the displacement values, and their peak stress values are shown in Figure 3. Similarly, the minimum peak stress value was observed under the −60° fixed model. One slight difference is that parallel K-wires did not perform better than dispersed wires in all results at this angle. For rotational conditions at −60°, the peak stress value of the K-wire for dispersed wires was 40.293 MPa, corresponding to 41.155 MPa for the K-Wire. However, the results are similar for both.

## Discussion

The evaluation of fracture fixation mainly includes clinical research and *in vitro* experimental simulation at present, and the difference in models can be effectively studied through *in vitro* experiments and computer modeling and simulation. Compared with clinical research, the uncertainty of fracture types in clinical patients’ experiments can be eliminated *in vitro*. In the fixation of distal humeral fractures, many scholars have studied the difference between K-wires and other fixation methods. However, the research mainly focused on the fixation of K-wires and did not involve sagittal plane studies in *in vitro* experiments. Bloom et al. (12) studied the optimal layout and number of K-wires for fixation of distal humeral fractures by 40 artificially synthesized pediatric humeral models, and believed that the layout and the dispersion of K-wires as much as possible at the fracture line would increase the stability of fixation. Schlitz et al. (13) studied the fixation effect of K-wires and screws through *in vitro* experiments and believed that the performance of screws fixation was better than K-wires. Franks et al. (14) also reached this conclusion. It cannot be denied that there are a large number of cartilage structures in the distal humerus of children, which have not been considered in *in vitro* experiments. Cartilage structures can be simulated in finite element studies. Kamara et al. (10) included this cartilage structure in the study of humeral condyle fractures. In the study of FEA of distal humeral fractures, Jeon et al. (9) believed that under the layout of a single K-wire, optimal biomechanical performance was achieved at an angle of 5°, while two wires were at 5°–45° or 5°–60°.

In our research, we observed and recorded the maximum displacement of the fracture block, and maximum von Mises stress of the bone and K-wires. The maximum displacement of the fracture block reflects the stability of the fixation mode under the load. The results showed the displacement distribution under tensile and rotational loads, and we found the minimum displacement value in the −60° model. Compared to other angles, this angle exhibits a turning point at 0°, where positive angles resulted in a worse fixation effect, while negative angles increased the stability of the fixation. This may be related to the fact that the humeral head in the sagittal plane is not on the same axis as the long axis of the humerus, but a more definitive conclusion requires further research. The maximum stress of the K-wires reflects the changes in stress the wire experiences. In our experiments, the maximum stress was concentrated near the fracture line. In the available literature, there are very few cases of K-wire breakage in this area, and all our results were below the yield stress of titanium alloy metal. The maximum stress of the bone is considered an indicator of whether a fracture is easily healed. The smaller the stress on the bone, the less stress the bone receives in this area; this is beneficial to healing to a certain extent. Our results showed that under the −60° condition, the internal fixation and maximum stress of the bone both reached their minimum values. Therefore, fixation at this angle is also most beneficial to fracture healing. However, the difference between parallel and dispersed K-wire placement was not reflected in this study, possibly maybe our scattered angle was small, as larger angles can still be achieved at −60°. On the other hand, the von Mises stress of the bone in the −60° model was still better than that of the other angles of fixation despite the difference in values in the parallel or diverging configurations. It is believed that the model under this angle can better promote the healing of the fracture and have less impact on the bone.

Jeon et al. (9) used one K-wire to study the angle in the coronal plane, and the optimal biomechanical performance was found to be at 5°. Afterward, the difference in the dispersed angle between two needles and three needles was studied. Our results showed that −60° is appropriate in the sagittal plane. In surgical K-wire placement for distal humeral fractures, the operative area and ideal number of K-wires is limited. Sagittal plane K-wire placement has not been extensively studied. In our surgical experience, the placement of the first needle is particularly critical, and the recommended 0° by the guidelines is close to the 5° recommended by Jeon et al. Whether the K-wire placement on the coronal plane is dispersed or parallel, the position of K-wire placement on the sagittal plane needs to be considered. Our results showed that −60° was optimal, but the results were close at negative angles and significantly better than positive angles. Therefore, we recommend that the entry point of the second K-wire be located behind and below the first K-wire.

In surgical treatment, anatomical reduction and compression of the epiphysis are essential. Both percutaneous closed reduction and open reduction internal fixation have satisfactory results. Exposed K-wires are a safe and economical choice when repairing lateral condylar fractures in children (15). For larger fracture fragments, good results can also be achieved by screw fixation (16). In addition to surgical treatment, satisfactory results can also be achieved through closed reduction for children (17). Studies have suggested that non-surgical treatment can be used for fractures with no displacement less than 2 mm (18). Regular follow-up with x-rays is necessary. A 10-year single-center study also confirmed this conclusion (19). Xie et al. (20) found that different classifications guide different fracture fixation methods. Our model has established a Milch type II fracture classification, and further discussion is needed for research on other fracture types.

## Limitations

There were some limitations to this study. First, both *in vitro* experiments and FEA are simulation for the real situation. Our model includes structures such as cartilage and ossification nucleus, but it still cannot achieve complete identity with reality. In order to avoid the influence of different opening angles on the experiment, the opening angle used in all dispersed pin configurations is the same as that when the opening angle is 0°. The purpose of this study is to investigate trends, and the precise motion of the load was not simulated. Future research should detail the precise loads of muscles and accurate motion, which will be applied to specific clinical practices.

## Conclusions

In summary, we created a pediatric lateral humeral condyle fracture model that includes cartilage and FEA to investigate the mechanical properties of K-wires with different sagittal plane angles for stabilizing fractures. The best mechanical performance was achieved when the sagittal plane angle was −60°; this angle of dispersed pinning was found to be superior to parallel pinning. It is important to consider clinical factors when applying these findings in practice.

## Data Availability

The raw data supporting the conclusions of this article will be made available by the authors, without undue reservation.
